# Survival factors in lung cancer patients with mutations in the *PROS1*, *SERPINC1*, *F2*, and *F5* genes

**DOI:** 10.18632/oncotarget.28914

**Published:** 2026-11-02

**Authors:** Ryan Brownlee, Aditya Birla, Hung Bui, Raniya Ahmad, Dongyu Zhang, Andrew Ji, Girindra Raval

**Affiliations:** ^1^The Medical College of Georgia at Augusta University, Augusta, GA 30912, USA; ^2^Department of Medicine, Division of Hematology and Oncology, Medical College of Georgia at Augusta University, Augusta, GA 30912, USA

**Keywords:** F5, PROS1, SERPINC1, lung cancer, thrombophilia

## Abstract

Purpose: Thrombophilia genes contribute to tumorigenesis and thrombotic events in lung cancer, yet current treatments do not incorporate gene-specific strategies for *F2*, *F5*, *PROS1*, and *SERPINC1*. We evaluated survival in lung cancer patients with thrombophilia mutations.

Materials and Methods: We queried TCGA-LUAD and TCGA-LUSC for primary lung cancers with *F2*, *F5*, *PROS1*, or *SERPINC1* mutations. STAR RNA-seq expression was included in univariate and multivariate Cox models comparing patients with versus without thrombophilia mutations, adjusting for age, race, AJCC stage, primary site, treatment type, laterality, and treatment modalities. Hazard ratios with 95% confidence intervals were calculated.

Results: We identified 569 patients with thrombophilia mutations and 850 without. Across all mutations, higher pathologic stage and right-sided tumors were associated with worse survival.

*F5* mutations were significantly associated with survival in middle-lobe (HR 1.49, *p* = 0.028), pleural (HR 1.33, *p* = 0.006), and upper-lobe (HR 1.20, *p* = 0.025) cancers. *SERPINC1* and *PROS1* mutations were associated with improved survival in upper-lobe cancers. Among upper-lobe cancers, African American patients with *F5* mutations had worse survival by age at diagnosis (HR 1.01, *p* = 0.015) and race (HR 1.33, *p* = 0.024).

Conclusions: Thrombophilia mutations influence lung cancer survival and may help refine gene-targeted therapies.

## INTRODUCTION

Lung cancer is a highly invasive cancer that metastasizes rapidly, remaining the second-most common cancer overall in both men and women [1]. Currently, lung cancer is responsible for nearly a quarter of all cancer deaths in the United States and globally [2, 3]. Despite advancements in therapeutic efficacy, lung cancer continues to have a poor prognosis, with a 5-year relative survival rate of just 19% in the United States [4]. This dismal outcome stems largely from late-stage presentation: only about 20% of cases are detected at a localized stage (stage I), for which the 5-year survival is approximately 57% [4]. Low-dose CT (LDCT) screening remains the only modality proven to reduce lung cancer mortality—data from the National Lung Screening Trial demonstrated a 20% relative reduction in lung cancer–specific deaths with three annual LDCT screens versus chest radiography [5]. Current U.S. Preventive Services Task Force guidelines recommend annual LDCT screening for adults 50–80 years of age with a ≥20 pack-year smoking history who currently smoke or have quit within the past 15 years [6]. Screening is not used in low-risk populations and, therefore, prognostic and screening gene markers will be key in reducing mortality for these groups.

Broadly, lung cancer is divided by either small cell or non-small cell lung cancers. Small cell lung cancer (SCLC) represents approximately 15% of all lung cancer diagnoses, and is characterized by rapid growth, early metastasis, and strong association with paraneoplastic syndromes such as Ectopic Cushing Syndrome or Syndrome of Inappropriate Anti-Diuretic Hormone [7]. Non-small cell lung cancers (NSCLC) represent 85% of lung cancer diagnoses, and include subtypes such as adenocarcinoma, squamous cell carcinoma, large cell carcinomas, and carcinoid tumors. These tumors all differ in primary location in the lung, as well as association with patient smoking history and genetic markers.

Genetic markers can provide insight into lung cancer pathophysiology and etiology. Genes involved in coagulation, such as *F2*, *F5*, *PROS1*, and *SERPINC1*, are of increasing interest in lung cancer research. The *F2* gene encodes prothrombin, the precursor to thrombin in the coagulation cascade. In relation to lung cancer, a study by Luo et al. found that the *F2* gene was significantly overexpressed in lung adenocarcinoma tumors compared to neighboring normal tissue [8]. The study found that a positive correlation between F2 gene expression and increased migration of regulatory T-cells, neutrophils, and mast cells, which are known mechanisms by which cancer cells create an immunosuppressive microenvironment. Another study by Wang et al. found that thrombin binds to and cleaves EGFR, activating the EGFR/AKT/mTOR signaling pathway and potentially promoting cell proliferation, migration, and chemotherapy resistance [9]. Thrombin activity has also been implicated in tumor growth, angiogenesis, and metastasis through its effects on protease-activated receptors and platelet activation [10]. Ultimately, these studies suggest that aberrant *F2* gene mutation and subsequently, thrombin activity are implicated in tumor growth, angiogenesis, and metastasis through its effects on protease-activated receptors and platelet activation.

The *F5* gene encodes coagulation Factor V, which serves as a cofactor in the prothrombinase complex. A condition resulting from mutation in this gene, Factor V Leiden, leads to resistance to activated Protein C and predisposes carriers to venous thromboembolism. Cancer-associated thrombosis is a major complication in oncology, as cancer is considered a hypercoagulable physiologic state, and may have an impact on patient’s prognosis [11]. Furthermore, a study by Tinholt et al. found a positive correlation between F5 gene expression and cellular infiltration in breast cancer [12]. Although not directly related to lung cancer, this finding could suggest a relationship between *F5* overexpression and the creation of lung cancer microenvironments. Therefore, dysregulation in this gene may influence tumor progression and metastatic potential.

The *PROS1* gene encodes Protein S, a cofactor for activated Protein C which inhibits factors V and VIII in the coagulation cascade. Therefore, PROS1 contributes to natural anti-coagulant regulation. Interestingly, a study by Suleiman et al. found that Protein S was significantly overexpressed in lung cancer cells and may play an additional role in tumorigenesis [13]. The study found that Protein S binds to TAM receptors (Tyro3 and MerTK) and activates the Erk, PKB/Akt, p38, and FAK pathways, which are key players in cancer growth, survival, and migration. These studies suggest that a Protein S mutation can disturb the balance between coagulation and fibrinolysis, with potential implications in the generation of tumor-friendly microenvironment in the body, as well as immune system evasion in malignancy.

Likewise, *SERPINC1*, which encodes antithrombin III, serves a crucial inhibitory role in the coagulation cascade and inflammation. A study by Zhang et al. found a correlation between high SERPINC1 expression and worse overall survival in lung cancer patients [14]. The study found that *in vitro*, the *SERPINC1* gene was involved in the activation of the PI3K/AKT pathway, which resulted in increased cancer growth, microenvironment formation, immunosuppression, and metastasis.

Mutations in *F2*, *F5*, *PROS1*, and *SERPINC1* are all clinically linked to thrombotic disorders, including prothrombin G20210A mutation, Factor V Leiden, Protein S deficiency, and antithrombin deficiency, respectively. However, more importantly, these prothrombotic states may contribute to hypercoagulability observed in malignancies, suggesting a dual role in thrombosis and tumor progression. The goal of our research is to assess significant factors in lung cancer outcomes relating to location, age, race, AJCC cancer staging, and treatment modalities among other factors in patients with mutated *F2*, *F5*, *PROS1*, and *SERPINC1* genes.

## RESULTS

Overall, coagulation-related gene expression demonstrated greater independent prognostic relevance in the comparison cohort than in the mutation cohort. In adjusted analyses, *PROS1* and *SERPINC1* were associated with improved overall survival in the comparison cohort, whereas *F2* and *F5* were associated with worse overall survival. In the mutation cohort, none of the evaluated gene counts remained statistically significant after adjustment, although *F2* and *F5* showed borderline associations with worsened survival. Across models, advanced disease stage and select anatomic tumor sites, particularly pleura, upper lobe of lung location, and middle lobe of lung location, were consistently associated with worse survival.

### Univariate Cox proportional hazards analysis

In univariate Cox proportional hazards analyses, the association between coagulation-related gene expression and overall survival differed by cohort. In the comparison cohort, higher *F2* gene counts were associated with worse overall survival (HR 1.15, 95% CI 1.09 to 1.21, *p* < 0.001), as were higher *F5* gene counts (HR 1.07, 95% CI 1.03 to 1.11, *p* < 0.001). In contrast, *PROS1* and *SERPINC1* were not significantly associated with overall survival in the comparison cohort (Table 1). In the mutation cohort, higher *F5* gene counts were associated with worse overall survival (HR 1.05, 95% CI 1.02 to 1.09, *p* = 0.003), while *F2* demonstrated a borderline association that did not meet statistical significance (HR 1.06, 95% CI 1.00 to 1.13, *p* = 0.053). *PROS1* and *SERPINC1* were not significantly associated with survival in the mutation cohort (Table 2).

**Table 1:** Univariate Cox proportional hazards analysis of gene mutations in the comparison group

Characteristic	HR	95% CI	*p*-value
*PROS1*	1.02	[0.97, 1.08]	0.4
*SERPINC1*	0.92	[0.83, 1.03]	0.14
*F2*	1.15	[1.09, 1.21]	1.60E-07
*F5*	1.07	[1.03, 1.11]	0.0004954

Survival analysis of gene expression in comparison group (Univariable).

**Table 2:** Univariate Cox proportional hazards analysis of gene mutations in the mutation group

Characteristic	HR	95% CI	*p*-value
*PROS1*	0.99	[0.94, 1.05]	0.7
*SERPINC1*	0.97	[0.88, 1.06]	0.5
*F2*	1.06	[1.00, 1.13]	0.053
*F5*	1.05	[1.02, 1.09]	0.003

Survival analysis of gene expression in patients with respective gene mutation (Univariable).

### Multivariate Cox proportional hazards analysis

#### PROS1

In the multivariable model evaluating *PROS1*, higher gene counts were independently associated with improved overall survival in the comparison cohort (HR 0.94, 95% CI 0.88 to 0.99, *p* = 0.025) (Table 3). This association was not observed in the mutation cohort, where *PROS1* gene counts were not significantly associated with survival after adjustment (HR 0.99, 95% CI 0.94 to 1.05, *p* = 0.7) (Table 4).

**Table 3:** Multivariate Cox proportional hazards analysis of survival factors in the *PROS1* comparison group

Characteristic	HR	95% CI	*p*-value
Age_at_diagnosis	1	1.00, 1.01	0.3
Gender			
Female	—	—	
Male	1.09	0.94, 1.27	0.2
Race_new			
White	—	—	
Black or african american	1.06	0.78, 1.45	0.7
Other	0.84	0.65, 1.10	0.2
Stage			
Stage I	—	—	
Stage II	1.45	1.20, 1.77	<0.001
Stage III	1.97	1.61, 2.40	<0.001
Stage IV	1.51	1.14, 2.01	0.004
Tissue_or_organ_of_origin			
Lower lobe, lung	—	—	
Lung, NOS	1.05	0.67, 1.65	0.8
Middle lobe, lung	1.46	0.96, 2.20	0.076
Pleura, NOS	1.73	1.36, 2.19	<0.001
Upper lobe, lung	1.28	1.06, 1.54	0.009
Treatment_type2			
Chemotherapy	—	—	
Ancillary Treatment	1.41	0.76, 2.62	0.3
Immunotherapy (Including Vaccines)	2.26	0.83, 6.16	0.11
Pleurodesis, NOS	1.09	0.79, 1.51	0.6
Radiation	1.13	0.95, 1.35	0.2
Targeted Therapy	1.1	0.91, 1.33	0.3
Laterality			
Left	—	—	
Right	1.18	1.02, 1.38	0.026
Counts_*PROS1*	0.94	0.88, 0.99	0.025

Survival analysis of *PROS1* in comparison group (Multivariable).

**Table 4:** Multivariate Cox proportional hazards analysis of survival factors in the *PROS1* mutation group

Characteristic	HR	95% CI	*p*-value
Age_at_diagnosis	1	0.99, 1.01	>0.9
Gender			
Female	—	—	
Male	1.01	0.88, 1.18	0.8
Race_new			
White	—	—	
Black or African American	1.2	0.94, 1.53	0.2
Other	0.85	0.67, 1.07	0.2
Stage			
Stage I	—	—	
Stage II	1.89	1.59, 2.26	<0.001
Stage III	2.12	1.76, 2.56	<0.001
Stage IV	1.62	1.18, 2.22	0.003
Tissue_or_organ_of_origin			
Lower lobe, lung	—	—	
Lung, NOS	0.72	0.49, 1.06	0.1
Middle lobe, lung	1.34	0.94, 1.90	0.11
Upper lobe, lung	1.18	1.01, 1.39	0.034
Treatment_type2			
Chemotherapy	—	—	
Ancillary Treatment	1.55	0.49, 4.91	0.5
Immunotherapy (Including Vaccines)	2.41	0.76, 7.59	0.13
Pleurodesis, NOS	—	—	
Radiation	1.04	0.88, 1.23	0.6
Targeted Therapy	0.98	0.81, 1.18	0.8
Laterality			
Left	—	—	
Right	1.2	1.04, 1.39	0.014
Counts	0.99	0.94, 1.05	0.7

Survival analysis in *PROS1* mutation group (Multivariable).

In the comparison cohort, worse overall survival was also associated with stage II disease (HR 1.45, 95% CI 1.20 to 1.77, *p* < 0.001), stage III disease (HR 1.97, 95% CI 1.61 to 2.40, *p* < 0.001), and stage IV disease (HR 1.51, 95% CI 1.14 to 2.01, *p* = 0.004). Among tumor site variables, pleura, NOS was associated with worse survival (HR 1.73, 95% CI 1.36 to 2.19, *p* < 0.001), as was upper-lobe lung location (HR 1.28, 95% CI 1.06 to 1.54, *p* = 0.009). Right-sided laterality was also associated with worse survival (HR 1.18, 95% CI 1.02 to 1.38, *p* = 0.026) (Table 3).

In the mutation cohort, *PROS1* was not independently associated with survival, but stage and tumor location remained prognostic. Stage II (HR 1.89, 95% CI 1.59 to 2.26, *p* < 0.001), stage III (HR 2.12, 95% CI 1.76 to 2.56, *p* < 0.001), and stage IV disease (HR 1.62, 95% CI 1.18 to 2.22, *p* = 0.003) were associated with worse survival. Upper-lobe lung location was also associated with worse survival (HR 1.18, 95% CI 1.01 to 1.39, *p* = 0.034), as was right-sided laterality (HR 1.20, 95% CI 1.04 to 1.39, *p* = 0.014) (Table 4).

#### SERPINC1

In the multivariable model evaluating *SERPINC1*, higher gene counts were independently associated with improved overall survival in the comparison cohort (HR 0.81, 95% CI 0.71 to 0.93, *p* = 0.002) (Table 5). In the mutation cohort, *SERPINC1* was not significantly associated with survival after adjustment (HR 0.99, 95% CI 0.94 to 1.05, *p* = 0.7) (Table 6).

**Table 5:** Multivariate Cox proportional hazards analysis of survival factors in the *SERPINC1* comparison group

Characteristic	HR	95% CI	*p*-value
Age_at_diagnosis	1	1.00, 1.01	0.3
Gender			
Female	—	—	
Male	1.1	0.95, 1.28	0.2
Race_new			
White	—	—	
Black or African American	1.24	0.90, 1.70	0.2
Other	0.85	0.65, 1.10	0.2
Stage			
Stage I	—	—	
Stage II	1.43	1.18, 1.74	<0.001
Stage III	1.85	1.51, 2.25	<0.001
Stage IV	1.42	1.07, 1.88	0.016
Tissue_or_organ_of_origin			
Lower lobe, lung	—	—	
Lung, NOS	1.03	0.66, 1.62	0.9
Middle lobe, lung	1.55	1.03, 2.35	0.036
Pleura, NOS	1.81	1.42, 2.30	<0.001
Upper lobe, lung	1.32	1.10, 1.59	0.003
Treatment_type2			
Chemotherapy	—	—	
Ancillary Treatment	1.35	0.73, 2.50	0.3
Immunotherapy (Including Vaccines)	2.41	0.88, 6.59	0.086
Pleurodesis, NOS	1.09	0.79, 1.50	0.6
Radiation	1.15	0.97, 1.36	0.12
Targeted Therapy	1.11	0.92, 1.35	0.3
Laterality			
Left	—	—	
Right	1.17	1.01, 1.36	0.038
Counts_*SERPINC1*	0.81	0.71, 0.93	0.002

Survival analysis of *SERPINC1* in comparison group (Multivariable).

**Table 6:** Multivariate Cox proportional hazards analysis of survival factors in the *SERPINC1* mutation group

Characteristic	HR	95% CI	*p*-value
Age_at_diagnosis	1	0.99, 1.01	>0.9
Gender			
Female	—	—	
Male	1.01	0.88, 1.18	0.8
Race_new			
White	—	—	
Black or African American	1.2	0.94, 1.53	0.2
Other	0.85	0.67, 1.07	0.2
Stage			
Stage I	—	—	
Stage II	1.89	1.59, 2.26	<0.001
Stage III	2.12	1.76, 2.56	<0.001
Stage IV	1.62	1.18, 2.22	0.003
Tissue_or_organ_of_origin			
Lower lobe, lung	—	—	
Lung, NOS	0.72	0.49, 1.06	0.1
Middle lobe, lung	1.34	0.94, 1.90	0.11
Upper lobe, lung	1.18	1.01, 1.39	0.034
Treatment_type2			
Chemotherapy	—	—	
Ancillary Treatment	1.55	0.49, 4.91	0.5
Immunotherapy (Including Vaccines)	2.41	0.76, 7.59	0.13
Pleurodesis, NOS	—	—	
Radiation	1.04	0.88, 1.23	0.6
Targeted Therapy	0.98	0.81, 1.18	0.8
Laterality			
Left	—	—	
Right	1.2	1.04, 1.39	0.014
Counts	0.99	0.94, 1.05	0.7

Survival analysis in *SERPINC1* mutation group (Multivariable).

In the comparison cohort, stage remained strongly associated with survival. Stage II (HR 1.43, 95% CI 1.18 to 1.74, *p* < 0.001), stage III (HR 1.85, 95% CI 1.51 to 2.25, *p* < 0.001), and stage IV disease (HR 1.42, 95% CI 1.07 to 1.88, *p* = 0.016) were each associated with worse overall survival. Several specific tumor sites were also associated with inferior survival, including middle lobe lung location (HR 1.55, 95% CI 1.03 to 2.35, *p* = 0.036), pleura, NOS (HR 1.81, 95% CI 1.42 to 2.30, *p* < 0.001), and upper-lobe lung location (HR 1.32, 95% CI 1.10 to 1.59, *p* = 0.003). Right-sided laterality was also associated with worse survival (HR 1.17, 95% CI 1.01 to 1.36, *p* = 0.038) (Table 5).

In the mutation cohort, *SERPINC1* did not retain independent prognostic significance. However, stage II (HR 1.89, 95% CI 1.59 to 2.26, *p* < 0.001), stage III (HR 2.12, 95% CI 1.76 to 2.56, *p* < 0.001), and stage IV disease (HR 1.62, 95% CI 1.18 to 2.22, *p* = 0.003) were associated with worse survival. Upper lobe lung location (HR 1.18, 95% CI 1.01 to 1.39, *p* = 0.034) and right-sided laterality (HR 1.20, 95% CI 1.04 to 1.39, *p* = 0.014) were also associated with worse survival (Table 6).

#### F2

In the multivariable model evaluating *F2*, higher gene counts were independently associated with worse overall survival in the comparison cohort (HR 1.16, 95% CI 1.10 to 1.23, *p* < 0.001) (Table 7). In the mutation cohort, *F2* gene counts did not meet statistical significance after adjustment, although the association approached significance (HR 1.07, 95% CI 1.00 to 1.14, *p* = 0.051) (Table 8).

**Table 7:** Multivariate Cox proportional hazards analysis of survival factors in the *F2* comparison group

Characteristic	HR	95% CI	*p*-value
Age_at_diagnosis	1.01	1.00, 1.02	0.14
Gender			
Female	—	—	
Male	1.12	0.96, 1.31	0.14
Race_new			
White	—	—	
Black or African American	1.11	0.81, 1.50	0.5
Other	0.89	0.68, 1.16	0.4
Stage			
Stage I	—	—	
Stage II	1.4	1.15, 1.71	<0.001
Stage III	1.85	1.51, 2.25	<0.001
Stage IV	1.4	1.06, 1.86	0.02
Tissue_or_organ_of_origin			
Lower lobe, lung	—	—	
Lung, NOS	1.13	0.72, 1.77	0.6
Middle lobe, lung	1.69	1.12, 2.56	0.013
Pleura, NOS	1.77	1.41, 2.23	<0.001
Upper lobe, lung	1.3	1.08, 1.56	0.006
Treatment_type2			
Chemotherapy	—	—	
Ancillary Treatment	1.17	0.63, 2.18	0.6
Immunotherapy (Including Vaccines)	2.17	0.80, 5.91	0.13
Pleurodesis, NOS	1.05	0.76, 1.44	0.8
Radiation	1.09	0.91, 1.29	0.4
Targeted Therapy	1.05	0.86, 1.27	0.7
Laterality			
Left	—	—	
Right	1.16	1.00, 1.35	0.047
Counts_*F2*	1.16	1.10, 1.23	<0.001

Survival analysis of *F2* in comparison group (Multivariable).

**Table 8:** Multivariate Cox proportional hazards analysis of survival factors in the *F2* mutation group

Characteristic	HR	95% CI	*p*-value
Age_at_diagnosis	1	0.99, 1.01	>0.9
Gender			
Female	—	—	
Male	0.96	0.78, 1.18	0.7
Race_new			
White	—	—	
Black or African American	1.06	0.79, 1.41	0.7
Other	1.39	0.98, 1.96	0.065
Stage			
Stage I	—	—	
Stage II	1.85	1.46, 2.35	<0.001
Stage III	2.19	1.68, 2.84	<0.001
Stage IV	1.6	1.02, 2.50	0.04
Tissue_or_organ_of_origin			
Lower lobe, lung	—	—	
Lung, NOS	0.88	0.53, 1.45	0.6
Middle lobe, lung	1.33	0.81, 2.19	0.3
Upper lobe, lung	1.2	0.97, 1.49	0.092
Treatment_type2			
Chemotherapy	—	—	
Ancillary Treatment	1.11	0.26, 4.79	0.9
Immunotherapy (Including Vaccines)	1.5	0.47, 4.83	0.5
Pleurodesis, NOS	—	—	
Radiation	0.86	0.68, 1.09	0.2
Targeted Therapy	0.86	0.66, 1.12	0.3
Laterality			
Left	—	—	
Right	1.24	1.01, 1.51	0.04
Counts	1.07	1.00, 1.14	0.051

Survival analysis in *F2* mutation group (Multivariable).

In the comparison cohort, worse overall survival was associated with stage II disease (HR 1.40, 95% CI 1.15 to 1.71, *p* < 0.001), stage III disease (HR 1.85, 95% CI 1.51 to 2.25, *p* < 0.001), and stage IV disease (HR 1.40, 95% CI 1.06 to 1.86, *p* = 0.02). Several tumor sites were associated with worse survival, including middle lobe lung location (HR 1.69, 95% CI 1.12 to 2.56, *p* = 0.013), pleura, NOS (HR 1.77, 95% CI 1.41 to 2.23, *p* < 0.001), and upper-lobe lung location (HR 1.30, 95% CI 1.08 to 1.56, *p* = 0.006). Right-sided laterality was also associated with worse survival (HR 1.16, 95% CI 1.00 to 1.35, *p* = 0.047) (Table 7).

In the mutation cohort, *F2* was not independently associated with survival after adjustment.

Stage remained the dominant prognostic feature, with stage II (HR 1.85, 95% CI 1.46 to 2.35, *p* < 0.001), stage III (HR 2.19, 95% CI 1.68 to 2.84, *p* < 0.001), and stage IV disease (HR 1.60, 95% CI 1.02 to 2.50, *p* = 0.04) associated with worse survival. Right-sided laterality was also associated with worse survival (HR 1.24, 95% CI 1.01 to 1.51, *p* = 0.04). Upper-lobe lung location demonstrated a trend toward worse survival but did not reach statistical significance (HR 1.20, 95% CI 0.97 to 1.49, *p* = 0.092) (Table 8).

#### F5

In the multivariable model evaluating *F5*, higher gene counts were independently associated with worse overall survival in the comparison cohort (HR 1.04, 95% CI 1.00 to 1.09, *p* = 0.032) (Table 9). In the mutation cohort, *F5* gene counts were not significantly associated with survival after adjustment (HR 1.03, 95% CI 1.00 to 1.07, *p* = 0.072) (Table 10).

**Table 9:** Multivariate Cox proportional hazards analysis of survival factors in the *F5* comparison group

Characteristic	HR	95% CI	*p*-value
Age_at_diagnosis	1	1.00, 1.01	0.3
Gender			
Female	—	—	
Male	1.11	0.95, 1.29	0.2
Race_new			
White	—	—	
Black or African American	1.13	0.83, 1.54	0.4
Other	0.83	0.64, 1.07	0.2
Stage			
Stage I	—	—	
Stage II	1.43	1.17, 1.74	<0.001
Stage III	1.85	1.51, 2.26	<0.001
Stage IV	1.46	1.10, 1.93	0.009
Tissue_or_organ_of_origin			
Lower lobe, lung	—	—	
Lung, NOS	1.06	0.68, 1.66	0.8
Middle lobe, lung	1.6	1.06, 2.43	0.027
Pleura, NOS	1.56	1.24, 1.96	<0.001
Upper lobe, lung	1.29	1.07, 1.55	0.006
Treatment_type2			
Chemotherapy	—	—	
Ancillary Treatment	1.41	0.76, 2.62	0.3
Immunotherapy (Including Vaccines)	2.24	0.82, 6.11	0.11
Pleurodesis, NOS	1.11	0.80, 1.53	0.5
Radiation	1.15	0.96, 1.36	0.12
Targeted Therapy	1.1	0.91, 1.33	0.3
Laterality			
Left	—	—	
Right	1.18	1.02, 1.37	0.03
Counts_*F5*	1.04	1.00, 1.09	0.032

Survival analysis of *F5* in comparison group (Multivariable).

**Table 10:** Multivariate Cox proportional hazards analysis of survival factors in the *F5* mutation group

Characteristic	HR	95% CI	*p*-value
Age_at_diagnosis	1.01	1.00, 1.02	0.015
Gender			
Female	—	—	
Male	1.03	0.90, 1.18	0.7
Race_new			
White	—	—	
Black or African American	1.33	1.04, 1.71	0.024
Other	0.84	0.66, 1.06	0.14
Stage			
Stage I	—	—	
Stage II	1.69	1.43, 1.99	<0.001
Stage III	1.9	1.60, 2.26	<0.001
Stage IV	1.67	1.31, 2.13	<0.001
Tissue_or_organ_of_origin			
Lower lobe, lung	—	—	
Lung, NOS	0.81	0.58, 1.14	0.2
Middle lobe, lung	1.49	1.04, 2.13	0.028
Pleura, NOS	1.33	1.08, 1.64	0.006
Upper lobe, lung	1.2	1.02, 1.41	0.025
Treatment_type2			
Chemotherapy	—	—	
Ancillary Treatment	1.46	0.83, 2.56	0.2
Immunotherapy (Including Vaccines)	2.02	0.75, 5.45	0.2
Pleurodesis, NOS	1.11	0.83, 1.48	0.5
Radiation	1.05	0.90, 1.22	0.6
Targeted Therapy	0.97	0.82, 1.15	0.7
Laterality			
Left	—	—	
Right	1.13	0.99, 1.28	0.072
Counts	1.03	1.00, 1.07	0.072

Survival analysis in *F5* mutation group (Multivariable).

In the comparison cohort, stage II (HR 1.43, 95% CI 1.17 to 1.74, *p* < 0.001), stage III (HR 1.85, 95% CI 1.51 to 2.26, *p* < 0.001), and stage IV disease (HR 1.46, 95% CI 1.10 to 1.93, *p* = 0.009) were associated with worse overall survival. Middle-lobe lung location (HR 1.60, 95% CI 1.06 to 2.43, *p* = 0.027), pleura, NOS (HR 1.56, 95% CI 1.24 to 1.96, *p* < 0.001), and upper-lobe lung location (HR 1.29, 95% CI 1.07 to 1.55, *p* = 0.006) were also associated with worse survival. Right-sided laterality was similarly associated with worse survival (HR 1.18, 95% CI 1.02 to 1.37, *p* = 0.03) (Table 9).

In the mutation cohort, *F5* gene counts did not retain statistical significance after multivariable adjustment. However, several clinical and tumor-related features were independently associated with worse survival. These included increasing age at diagnosis (HR 1.01, 95% CI 1.00 to 1.02, *p* = 0.015), Black or African American race (HR 1.33, 95% CI 1.04 to 1.71, *p* = 0.024), stage II disease (HR 1.69, 95% CI 1.43 to 1.99, *p* < 0.001), stage III disease (HR 1.90, 95% CI 1.60 to 2.26, *p* < 0.001), and stage IV disease (HR 1.67, 95% CI 1.31 to 2.13, *p* < 0.001). Tumor site was also prognostic, with middle lobe lung location (HR 1.49, 95% CI 1.04 to 2.13, *p* = 0.028), pleura, NOS (HR 1.33, 95% CI 1.08 to 1.64, *p* = 0.006), and upper lobe lung location (HR 1.20, 95% CI 1.02 to 1.41, *p* = 0.025) associated with worse overall survival. Right-sided laterality showed a borderline association but did not meet statistical significance (HR 1.13, 95% CI 0.99 to 1.28, *p* = 0.072) (Table 10).

## DISCUSSION

This is the first study to our knowledge to assess overall survival factors in mutated *F2*, *F5*, *PROS1*, and *SERPINC1* genes in lung cancer. *F5*, *SERPINC1*, and *PROS1* were significant primarily in the upper lobe of the lung. This finding may be attributed to the anatomical differences between the upper lobe and lower lobe of the lungs. Medical literature has found a non-uniform oxygenation levels throughout the lungs, with the upper lobe having the highest level of oxygenation. The ventilation-perfusion (V/Q) ratio for the apex of the lung is approximately 3.0, which can be explained by a decreased ventilation and a severely decreased perfusion [15, 16]. As a result, the upper lobe of the lungs has higher exposure to oxygen and lower blood perfusion. This difference predisposes the upper lobe to increased oxidative stress and cellular damage [16]. Mutations in *F5*, *SERPINC1*, and *PROS1* genes may contribute to further oxidative stress or the creation of hypoxic environments via thrombogenesis, ultimately leading to cellular damage and tumor genesis.

Given the role of these genes in modulating parts of the coagulation pathway and overall, coagulability, it may be plausible that the worse survival is accounted for by increased thrombotic events. Although this database does not account for these types of events, this should be considered, especially for loss of function mutations. Recent studies have shown venous thromboembolism to be a predictor of early mortality in non-small cell lung carcinoma patients [17]. Interestingly, younger lung cancer patients appear to be more at risk for thromboembolic events [18]. Future studies may want to explore these genes by age to further elucidate differences in survival.

Pathologic stage was consistently and strongly associated with worse survival in every gene.

Furthermore, different genetic mutations were more prevalent in tumors of different locations.

Tumors affecting the middle lobe, pleura, and upper lobe of the lung were more likely to express *F5* gene mutations. Comparatively, tumors affecting the upper lobe were also more likely to express mutations in *SERPINC1* and *PROS1*. Mutations in all genes studied, *SERPINC1*, *F5*, *F2*, *PROS1*, were more likely to yield a worse outcome when present in cancers of the right lung compared to the left lung.

Interestingly, unlike the other aforementioned genes involved in thrombotic disorders, *F5* mutations appear to disproportionally affect survival based off the patients’ ethnicity. Of particular concern, from the multivariate hazards analysis, *F5* is found to have a noticeably worse significant hazards ratio (HR 1.33, [1.04, 1.71] 95% CI) for African Americans within the mutation group in relation to the comparison group (HR 1.13, [0.83, 1.54] 95% CI). It is well researched that Factor V Leiden is most commonly found in patients of European Caucasian descent and less prevalent in African American patients, subsequently presenting as a lower risk gene mutation in the African American population for venous thromboembolism [19]. However, findings within our study illustrate the need for better understanding and investigation of the associated risks and mechanisms of *F5* mutations within African American populations, especially with consideration of the gene’s interplay with lung cancers.

Moreover, the *F5* gene, distinctive from the other genes, possesses a slightly worse hazards ratio (HR 1.01, [1.00, 1.02] 95% CI) for age of diagnosis that is statistically significant within the mutations group in contrast to the comparison group. In turn, the relation and severity of the negative impact of *F5* mutations on lung cancer patients may be dependent upon the patient’s age at diagnosis, leading to potentially age-differentiated rates of lung cancer survival within patients affected by *F5* mutations.

When considering all variables of interest, gene count is observed to be statistically significant within the comparison group of all genes of interest. On the other hand, this variable is found to be insignificant in all mutation groups of each gene. These trends are explained via the structure of the study. Since the study population is split between two groups based on the presence of a gene mutation, rather than specifically abnormally lower or higher expression of each thrombotic gene, it can be reasoned that gene count is insignificant within the mutation group due to the averaging of gain and loss-of-function mutations into a lesser extreme expression level. Within the comparison groups, however, the significance of gene count indicates the potential benefit of utilizing identified changes within these genes as a prognostic tool for lung cancers. Further investigation into specifically lower expression and loss-of-function mutations of these genes may be necessary, alongside their side effects.

### Clinical implications

The results of this study suggest that African American patients with an *F5* mutation may be at increased risk for worse outcomes in lung cancer. Prior studies have discovered unique mutations specific to these populations [20, 21]. However, no studies have looked at the *F5* gene. *F5* may serve as a future prognostic marker for this population and should be further studied. If future studies confirm this finding, loss of function mutations of the *F5* gene may require more aggressive or personalized treatment, especially in African American populations.

Factor V Leiden is an inherited condition caused by a loss of function in the *F5* gene. Currently, there are no disease specific guidelines for this population and there are no studies specifically studying this population’s risk of lung cancer. These patients could be at increased risk of worse outcomes and, therefore, should be considered for future studies. If future studies confirm this study’s findings, Factor V Leiden specific guidelines for lung cancer should be considered.

### Limitations

This study comes with limitations which should be considered. First, it is retrospective in nature and, therefore, many of the patient variables could not be controlled for such as the type and timing of treatment. Additionally, the study utilized STAR RNA expression counts for each gene. These readings represent a snapshot in time of the patient’s expression and could differ significantly throughout the progression of the cancer. Our study assessed metastasis by AJCC pathologic M which does not indicate the specific site. Site is a major prognostic factor and, therefore, future studies should include metastatic site in their analysis. Lastly, mutations were not separated by type. Therefore, our data included loss of function and gain of function and future studies should separate the two.

## MATERIALS AND METHODS

The Cancer Genome Atlas (TCGA) was queried for primary lung cancer patients from the TCGALUAD and TCGA-LUSC projects with mutations in either the *PROS1*, *SERPINC1*, *F2*, or *F5* genes.

Additionally, clinical data of a comparison group of all patients from the TCGA-LUAD and TCGALUSC projects were queried. All patients with missing or incomplete data for these variables were excluded. A total of 569 patients met the inclusion and exclusion data for this study. STAR RNA gene expression quantification data of each gene were collected for the mutated and comparison group to be used in this analysis. We compared outcomes for each gene between two groups: patients with mutated *PROS1*, *SERPINC1*, *F2*, or *F5* and patients without mutation of these genes. The primary outcomes were hazard ratios.

Patient age at diagnosis, race, AJCC pathologic stage, primary site, treatment type, and laterality data were pulled for the analysis. Treatment types included chemotherapy, ancillary treatment, immunotherapy (including vaccines), pleurodesis, radiation, and targeted therapy. All patients with missing or incomplete data for these variables were excluded. A total of 569 patients in the mutation group and 850 patients in the comparison group met the inclusion and exclusion criteria for this study.

Univariate and multivariate Cox Proportional Hazards Analyses were conducted to assess which variables were most associated with overall survival. Hazard ratios (HR) and 95% confidence intervals were calculated for the analysis.

## CONCLUSIONS

Higher pathologic stage is significantly associated with worse survival in all groups. *F5* mutations was protective in middle lobe, pleura, and upper lobe primary sites. *PROS1* and *SERPINC1* mutation were protective in upper lobe primary sites. African Americans with *F5* mutations experienced worse survival. Mutations in *F5*, *F2*, *SERPINC1*, and *PROS1* were all significantly associated with worse survival in right-sided lung cancer.
